# LncRNA TUG1 regulates the development of ischemia-reperfusion mediated acute kidney injury through miR-494-3p/E-cadherin axis

**DOI:** 10.1186/s12950-021-00278-4

**Published:** 2021-03-04

**Authors:** Li Chen, Jun-Ying Xu, Hong-Bao Tan

**Affiliations:** 1 Department of Nephrology, Brain Hospital of Hunan Province, Changsha, 410007 Hunan Province P.R. China; 2 Department of Anesthesiology, The Fourth Hospital of Changsha, No.70, Lushan Road, Yuelu District, Changsha, 410006 Hunan Province P.R. China

**Keywords:** Acute kidney injury, lncRNA TUG1, miR-494-3p, E-cadherin

## Abstract

**Background:**

Acute kidney injury (AKI) results from renal dysfunction caused by various causes, resulting in high mortality. The underlying mechanisms of ischemia-reperfusion (I/R) induced AKI is very complicated and needed for further research. Here, we sought to found out the functions of lncRNA TUG1 in I/R-induced AKI.

**Methods:**

In vivo model was constructed by I/R-induced mice and in vitro model was constructed by hypoxia/reoxygenation (H/R)-induced HK-2 cell. Kidney tissue damage was evaluated through H&E staining in mice. Cell flow cytometry was used to detect the degree of apoptosis. TUG1, miR-494-3p and E-cadherin were determined both by RT-PCR and western blot. Dual luciferase assay was employed to validate the relationships between TUG1, miR-494-3p and E-cadherin. Inflammatory factors including IL-1β, TNFɑ and IL-6 were evaluated by ELISA.

**Results:**

lncRNA TUG1 was decreased while miR-494-3p was elevated in vivo and in vitro. Overexpression of TUG1 or transfection with miR-494-3p inhibitor significantly alleviated cell apoptosis. MiR-494-3p directly targeted E-cadherin and TUG1 suppressed cell apoptosis via serving as a miR-494-3p sponge to disinhibit E-cadherin.

**Conclusion:**

lncRNA TUG1 alleviated I/R-induced AKI through targeting miR-494-3p/E-cadherin.

## Background

As a common clinical syndrome with high morbidity and mortality, AKI is characterized by sudden loss of kidney function [1, 2]. It has been guessed that AKI is related to robust inflammatory reaction, oxidative stress, intracellular Ca^2+^ overload, renin-angiotensin activation and microcirculation disturbance. I/R-induced damage is one of the most vital reason for AKI and develops during cardiac surgery [3], kidney transplantation [4], and renal vascular obstruction [5]. Even so, the underlying mechanisms of Ischemia-reperfusion induced AKI is very complicated and remain to be fully elucidated.

Long noncoding RNAs (lncRNAs) are RNAs longer than 200 nucleotides without protein coding ability [6, 7], which are transcribed by Pol II [8]. LncRNAs are vital in epigenetic regulation, gene transcription, gene translation and mRNA processing and regulate wide range of pathological processes [9, 10]. As well-known members of lncRNAs, plasmacytoma variant translocation (PVT1) and nuclear enriched abundant transcript 1 (NEAT1) could accelerate the progression of AKI. LPS-induced septic AKI was accelerated by PVT1 through JNK/NF-κB [11]. NEAT1 upregulation prompted LPS-induced AKI through negatively regulating miR-494-3p [12]. In addition, Liu et al. initially found lncRNA taurine up-regulated gene 1 (TUG1) was downregulated in sepsis AKI than that in control. Sepsis AKI may be relieved by upregulation of TUG1 through miR-142-3p/SIRT1 axis [13]. However, whether TUG1 was related with ischemia-reperfusion induced AKI remains unknown.

MicroRNAs are a series of small non-coding RNAs and bind to the 3′-untranslated regions of mRNAs to influence target gene and participate in cell growth, migration, apoptosis and differentiation [14, 15]. Emerging evidence indicates that lncRNAs can participate in the pathological process of AKI by regulating microRNAs [16]. miR-494 is transcribed by RNA polymerase II as part of capped and polyadenylated primary transcripts (pri-miRNAs) while miR-494-3p was generated from the precursor miRNA (pre-miRNA) which is a product of the pri-miRNAs cleaved by the Drosha ribonuclease III enzyme. Firstly known as elevated miRNA in human retinoblastoma tissues, miR-494 was used in cancer research [17]. Subsequent studies indicated that miR-494-3p is highly expressed in I/R-induced AKI and thus attenuated the kidney protective gene ATF3, leading to cell apoptosis and more aggravated kidney injury [14]. Since the expressed trend of TUG1 and miR-494-3p was opposite, we speculated whether lncRNA TUG1 and miR-494-3p are related in the regulation of I/R-induced AKI.

In our research, we want to found out the effect and mechanisms of lncRNA TUG1 in I/R-induced AKI. Therefore, we firstly evaluated the expressed level of TUG1 in kidney tissues and HK-2 cells. Subsequently, the roles of TUG1 overexpression on I/R-induced AKI mice and H/R-induced HK-2 cell model were examined, which may present a new strategy for I/R-induced AKI treatment.

## Materials and methods

### Animals

C57BL/6 J mice (*n* = 10, Male, 10–12 weeks, 20–25 g weight) were purchased from Hunan SJA Laboratory Animal Co., Ltd. (Changsha, China). Mice were maintained under a standard feeding environment with 12/12-h light/dark cycle and room temperature of 18–26 °C. Water and food were provided quantitatively. The Ethics Committee of Brain Hospital of Hunan Province approved all animal experimental protocols (No. K2019021).

### In vivo mice AKI model

Two groups (*n* = 5/group) including sham group and I/R group were randomly divided. Surgical procedure referred to the previous literature [18]. Pentobarbital (60 mg/kg; Sigma-Aldrich) was intraperitoneal injected into mice and a heating plate was used to maintain body temperature during surgery. In I/R group, the isolated bilateral renal pedicles were suffered for 30 min renal ischemia and removing clamp for reperfusion. In sham group, the standard surgery was conducted without clamping the bilateral renal pedicles. After reperfusion for 2 h, mice were sacrificed to collect blood, urine, and kidney tissue sample for further analysis.

### Histopathology

After fixing in 4% formalin for 24 h at 25 °C, the kidney tissues were embedded in paraffin, which was cut into 5 μm-thick paraffin section. Subsequently, hematoxylin-eosin staining was performed as previously described [19]. Tissue section viewed and photographed under microscope (Carl Zeiss MicroImaging GmbH, Göttingen, Germany). According to the research of Jaklien C. Leemans et al. [20], the tubular injury score was assessed by a 5-point scale with the occurrence of necrosis.

### Cell culture

Human kidney tubular epithelial cell line HK-2 was purchased from the institute of Biochemistry and cell Biology of the Chinese academy of Sciences (Shanghai, China). Dulbecco’s modified Eagle’s medium (DMEM; Invitrogen; Thermo Fisher Scientifc, Inc., Waltham,ma, USA) containing 10% fetal bovine serum (FBS; Invitrogen; Thermo fisher Scientific, Inc.) and 100 u/ml penicillin and 100 mg/ml streptomycin was used as standard culture condition. The cell incubator was maintained at 37 °C with saturation humidity and 5% CO_2_.

### Hypoxia/reoxygenation (H/R) cell model

The hypoxic condition was constructed in HK-2 cells maintaining in complete medium HypOxystation H35 (Don Whitley Scientific) for 2 days with 1% Oxygen, 5% CO_2_ and 94% N_2_ at 37 °C [21]. Then fresh medium was applied for additional 24 h referring to reoxygenation. During the re-oxygenation time, cells were transfected by constructed vectors or plasmids. Normal cells were cultured under standard conditions.

### Cell transfection

Using the PfuUltra II Fusion HS DNA Polymerase (Stratagene, Agilent Technologies, Santa Clara, CA, USA), the amplified TUG1 cDNA was inserted into pcDNA3.1 vector (Invitrogen) to construct the TUG1-overexpressed plasmid as pcDNA TUG1. Small interfering RNA targeting TUG1 (si-TUG1) and negative control (si-NC) were obtained from Qiagen GmbH (Hilden, Germany). Also, miR-494-3p mimics, miR-494-3p inhibitor and miR-NC, si-E-cad and si-NC were obtained from GenePharma (Shanghai, China). Aforementioned vectors or plasmids were transfected into cells using lipofectamine 2000 (Invitrogen). After 24 h transfection, cells were collected for experiments.

### Detection of renal functional parameters and inflammatory cytokines

Using a Beckman Autoanalyzer (Beckman Coulter), the level of serum creatinine (SCr) and blood urea nitrogen (BUN) were tested. An ELISA Kit from Cosmo Bio and an ELISA Kit from Sangon Biotech were respectively used to examine the urine concentration of Kim-1 and the cultured concentration of TNF-α, IL-6, and IL-1β.

### Quantitative reverse transcription polymerase chain reaction (qRT-PCR)

Kidney tissues and HK-2 cells were lysed to obtain total RNA using TRIzol reagent (Life Technologies Corporation, Florida). A TaqMan miRNA Reverse Transcription Kit (Applied Biosystems) was used to obtain cDNA. TaqMan Universal Master Mix II, TaqMan miRNA assays was carried out for miR-494-3p and U6 and TaqMan gene expression assays for TUG1 and GAPDH. Endogenous controls was set as U6 or GAPDH. Finally, the relative gene expression was determined by calculating the 2^-∆∆ct^ equation.

### Cell apoptosis

Cells (10,000 cells/well) were cultured in 96-well plate. After 24 h cells were collected through washing 3 times with PBS buffer. Then, FITC-annexin V (BD Biosciences, USA) and propidium iodide was added for 15 min incubation. The results were pictured by FACScan Flow Cytometer (BD Biosciences) and the content of apoptotic cells were summarized using Cellquest software (BD Biosciences).

### Western blot analysis

Determining by a BCA protein assay kit (Bio-Rad laboratories, inc., Hercules, CA, USA), the extracting total proteins from kidneys and HK-2 cells were loaded on sodium dodecyl sulfate (SDS) polyacrylamide gel electrophoresis (PAGE) gels for separation and then transferred to polyvinylidene fluoride (PVDF) membranes. Following 1 h skim milk (5%) incubation at room temperature, primary antibodies including Bcl-2 (ab59348, Abcam, Cambridge, MA, USA), Bax (ab32503, Abcam), cleaved caspase3 (ab49822, Abcam), total caspase3 (ab13847, Abcam), E-cadherin (CST, Danvers, MA, USA) and β-actin (cat. no. 8457, Cell Signaling Technology) were added respectively for overnight incubation at 4 °C. Secondary antibodies including goat anti-rabbit (ab205718, Abcam) and goat anti-mouse (ab6789, Abcam) were used for 2 h incubation at room temperature. The results were calculated through enhanced chemiluminescence reagents (Pierce, Rockford, IL, USA).

### Luciferase assay

The reporter vectors of TUG1-WT and TUG1-MUT or E-cadherin-WT and E-cadherin-MUT were constructed by inserting the cDNA fragments of TUG1 or E-cadherin containing the predicted binding site of miR-494-3p or mutated binding site of miR-494-3p into the pmirGlO Dual-luciferase miRNA Target Expression Vector (Promega, Madison, WI). HK-2 cells were culture in 96-well plates until the cell confluence was 50–70%. Using Lipofectamine 2000 (Invitrogen), cells were co-transfected with reporter vectors and miR-NC or miR-494-3p mimics. Following 48 h of post-transfection, the luciferase activity was estimated by Dual-Luciferase Reporter Assay System (Promega).

### Statistical analysis

All the experiments in this research were repeated three times independently (*n* = 3). Data were presented as the mean ± standard deviation. GraphPad Prism software (GraphPad Software, La Jolla, CA, USA) was used to analyzed the statistical significance between different groups using analysis of variance or a two tailed Student’s *t*-test. A statistically significant difference was indicated when *P* < 0.05.

## Results

### TUG1 expression was decreased both in I/R-induced mice and H/R-induced HK-2 cells

**Fig. 1:**
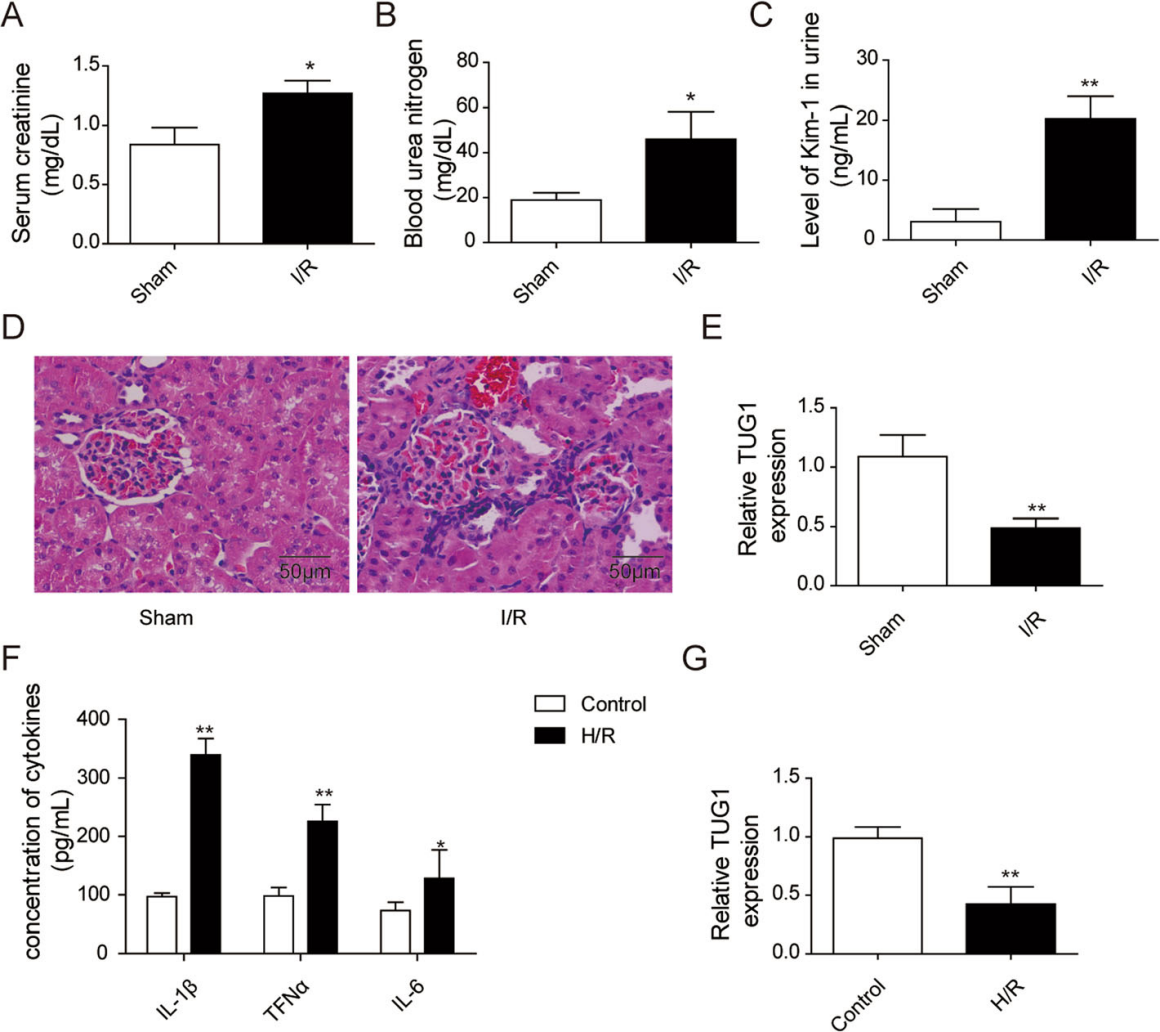
TUG1 expression was decreased both in I/R-induced mice and H/R-induced HK-2 cells. **a-c**: The levels of SCr, BUN and Kim-1 were detected via ELISA assay. **d**: Renal sections of mice were stained using H&E. **e**: The relative expression level of TUG1 in vivo was tested by qRT-PCR. **f**: The expression level of cytokines including TNF-α, IL-1β, and IL-6 were assessed by ELISA. **g**: The relative expression level of TUG1 in vitro was measured by qRT-PCR. Results were presented as mean ± SD. *P < 0.05, **P < 0.01

To investigate the abnormal gene expression of TUG1 in response to AKI, I/R-induced mice and H/R-induced HK-2 cells were used, respectively. Comparing to sham group serum levels of SCr (Fig. 1a) and BUN (Fig. 1b) were significantly upregulated in I/R group, SCr mainly depends on the glomerular filtration rate and BUN is the main component of non-protein nitrogen, accounting for half. When the serum level of Scr and Bun are obviously higher, the kidney function has been severely damaged. Simultaneously, the results of urine level of Kim-1 (Fig. 1c) and histopathological analysis (Fig. 1d) showed that I/R-induced mice kidney displayed tubular damage. Kim-1 is a transmembrane glycoprotein of renal proximal convoluted tubule epithelial cells. Its expression is significantly enhanced when the proximal convoluted tubule epithelial cells are regenerated after damage, serving as a reliable scientific marker for detecting early renal injury. Comparing with control group, the concentrations of inflammatory factors including IL-1β, TNFɑ and IL-6 were dramatically elevated in H/R-induced HK-2 cells (Fig. 1f). TUG1 was dramatically reduced in I/R group and H/R group (Fig. 1E and G). Taken together, TUG1 was significantly decreased in vivo and in vitro.

### Overexpression of TUG1 alleviated cell apoptosis caused by H/R

**Fig. 2:**
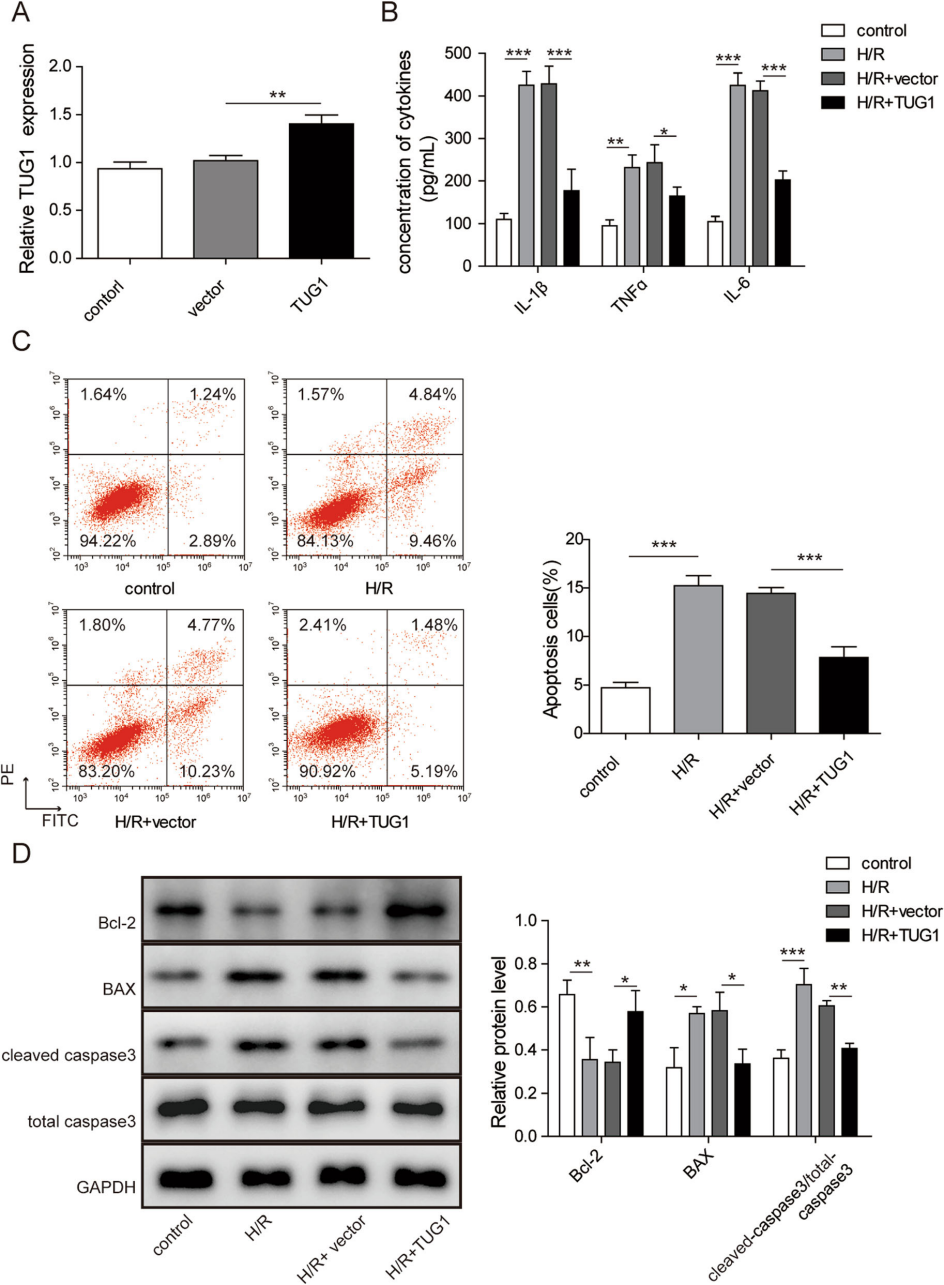
Overexpression of TUG1 alleviated cell apoptosis caused by H/R. **a**: The relative expression level of TUG1. **b**: The concentration of cytokines including TNF-α, IL-1β, and IL-6 were measured by ELISA. **c**: Cell apoptosis was assessed by flow cytometry. **d**: The relative expression levels of Bcl-2, Bax and cleaved-caspase-3 in H/R-induced HK-2 cells were detected by western blot. Results were presented as mean ± SD. *P < 0.05, **P < 0.01, ***P < 0.001

To further explore the role of TUG1, cells were transfected with pcDNA-TUG1 and empty vector, respectively. Figure 2a suggested that transfected cells could be used for experiment since the expression of TUG1 was higher in pcDNA-TUG1 group, demonstrating a successful transfection. The results of ELISA indicated that overexpressed TUG1 reduced the level of IL-1β, TNFɑ and IL-6 (Fig. 2b). The effect of TUG1 on cell apoptosis was detected by flow cytometry. Comparing with that of H/R + vector group, the number of apoptosis cells were reduced in the H/R + TUG1 group (Fig. 2c). Concurrently, the results of western blot displayed the same trend in apoptotic proteins expression such as Bcl-2, Bax, cleaved caspase3/total caspase3(Fig. 2d). Taken together, overexpression of TUG1 effectively alleviated cell apoptosis induced by H/R.

### TUG1 acted as a sponge of miR-494-3p

**Fig. 3:**
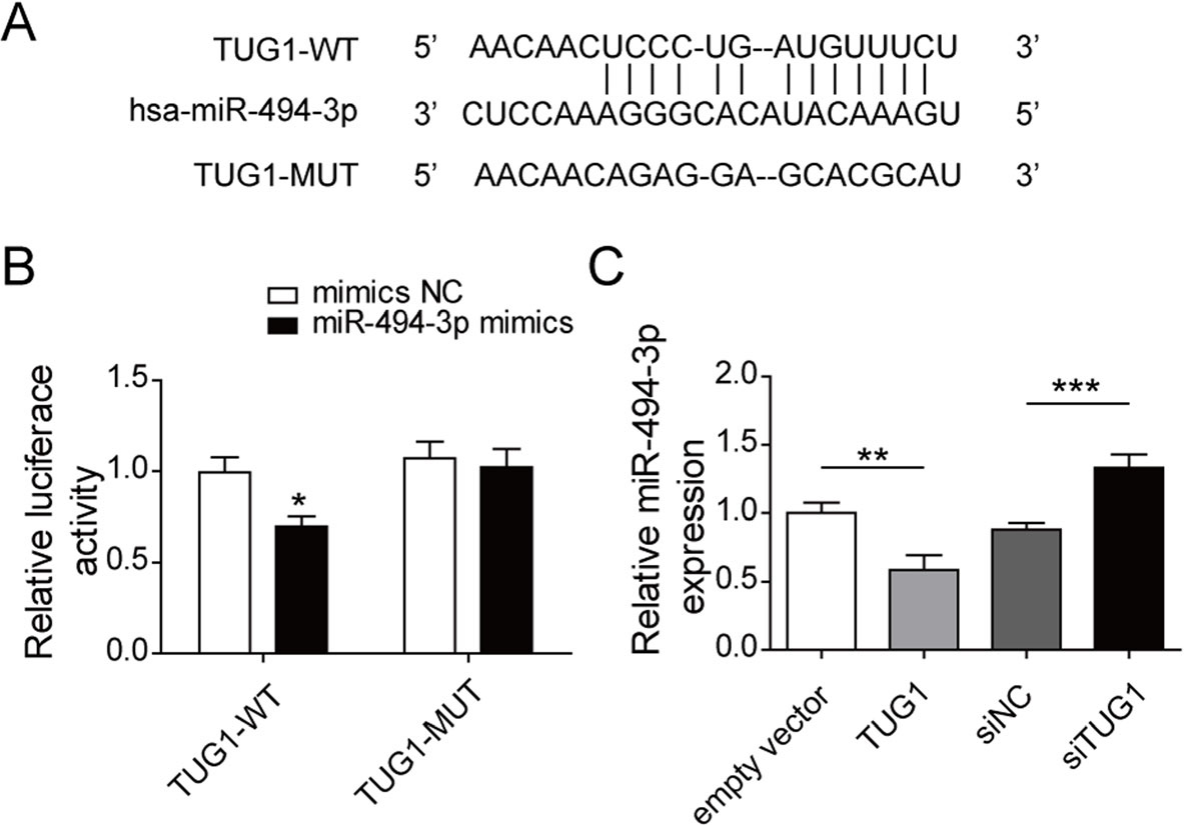
TUG1 acted as a sponge of miR-494-3p. **a**: The putative binding site of TUG1 and miR-494-3p. **b**: Relative luciferase activities was assessed by luciferase reporter assay. **c**: The relative expression of miR-494-3p was detected by qRT-PCR. Results were presented as mean ± SD. *P < 0.01, **P < 0.001

We further examined the possible mechanism by searching the potential targets of TUG1. The relationship between TUG1 and miR-494-3p was found out on lncBase Predicted v.2 bioinformatics tools (http://carolina.imis.athena-innovation.gr/). Figure 3a displayed the predicted binding sequences of TUG1 and miR-494-3p. Figure 3b of dual luciferase assay showed miR-494-3p mimics remarkably decreased the luciferase activity of TUG1. Moreover, the qRT-PCR results demonstrated comparing to the siNC group, TUG1 overexpressed dramatically reduced miR-494-3p in HK-2 cells relative to empty vector group and TUG1 inhibited increased miR-494-3p (Fig. 3c). Thus, we speculated miR-494-3p may be a target of TUG1 and TUG1 negatively affected miR-494-3p level.

### miR-494-3p participated in TUG1-mediated cell injury

**Fig. 4.**
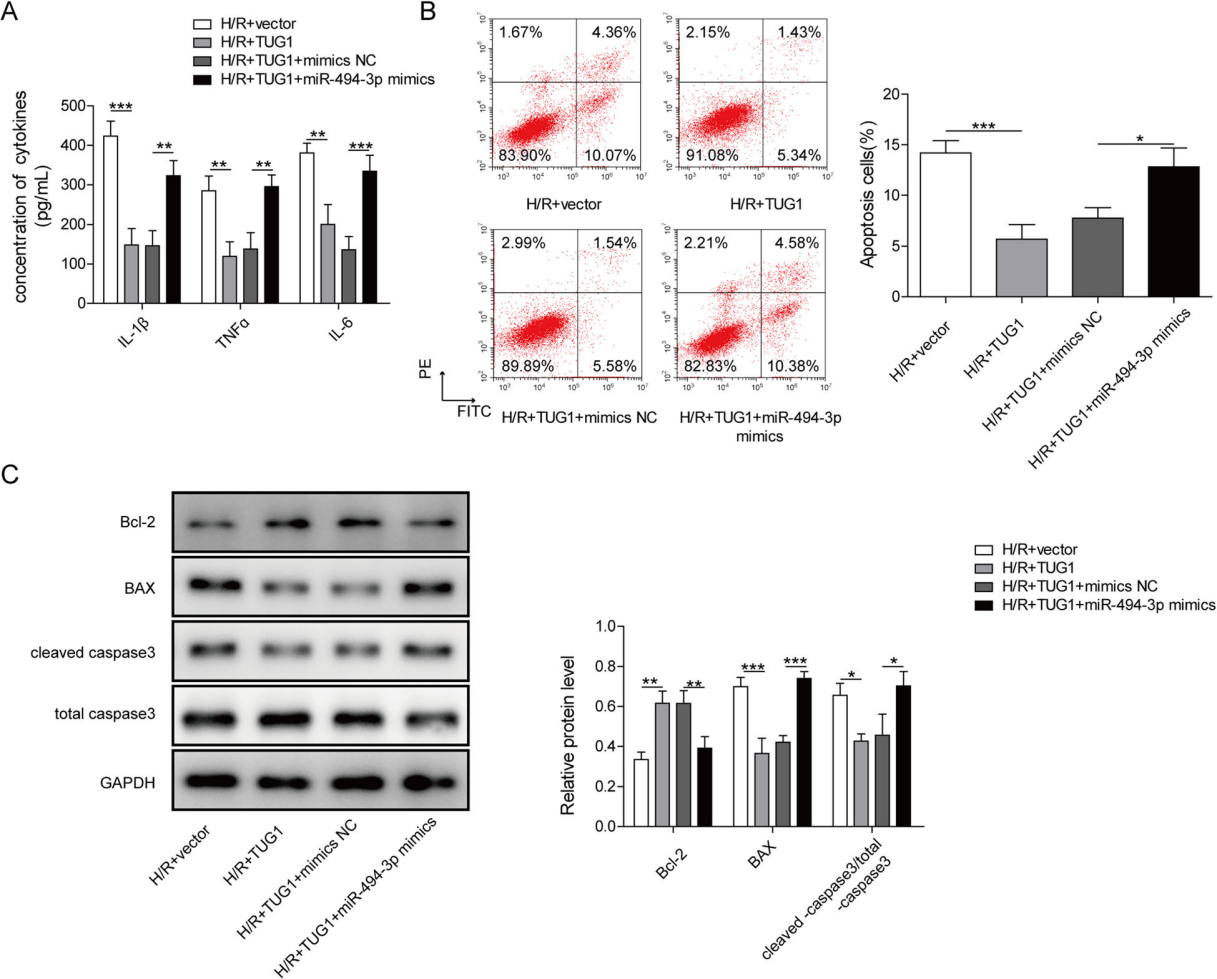
miR-494-3p participated in TUG1-mediated cell injury. **a**: The concentration of cytokines including TNF-α, IL-1β, and IL-6 were assessed by ELISA. **b**: Flow cytometry was used to analyze cell apoptosis. **c**: The relative expression levels of Bcl-2, Bax and cleaved-caspase-3 were measured by western blot. Results were presented as mean ± SD. *P < 0.05, **P < 0.01, ***P < 0.001.

To further verify whether TUG1 is related to cell injury induced by H/R through regulating miR-494-3p, miR-494-3p mimics and TUG1 were co-transfected into H/R-induced HK-2 cells to assess inflammatory response and cell apoptosis. Transfection with miR-494-3p mimics reversed the influences of TUG1 up-regulation on H/R-induced HK-2 cells by not only promoting the production of IL-1β, TNFɑ and IL-6 (Fig. 4a), but also developing cell apoptosis (Fig. 4b) and increasing Bax and cleaved-caspase3, as well as decreasing Bcl-2 (Fig. 4c). All these data indicated that TUG1 mediated H/R-induced HK-2 cell injury via miR-494-3p.

### MiR-494-3p targeted to E-cadherin

**Fig. 5:**
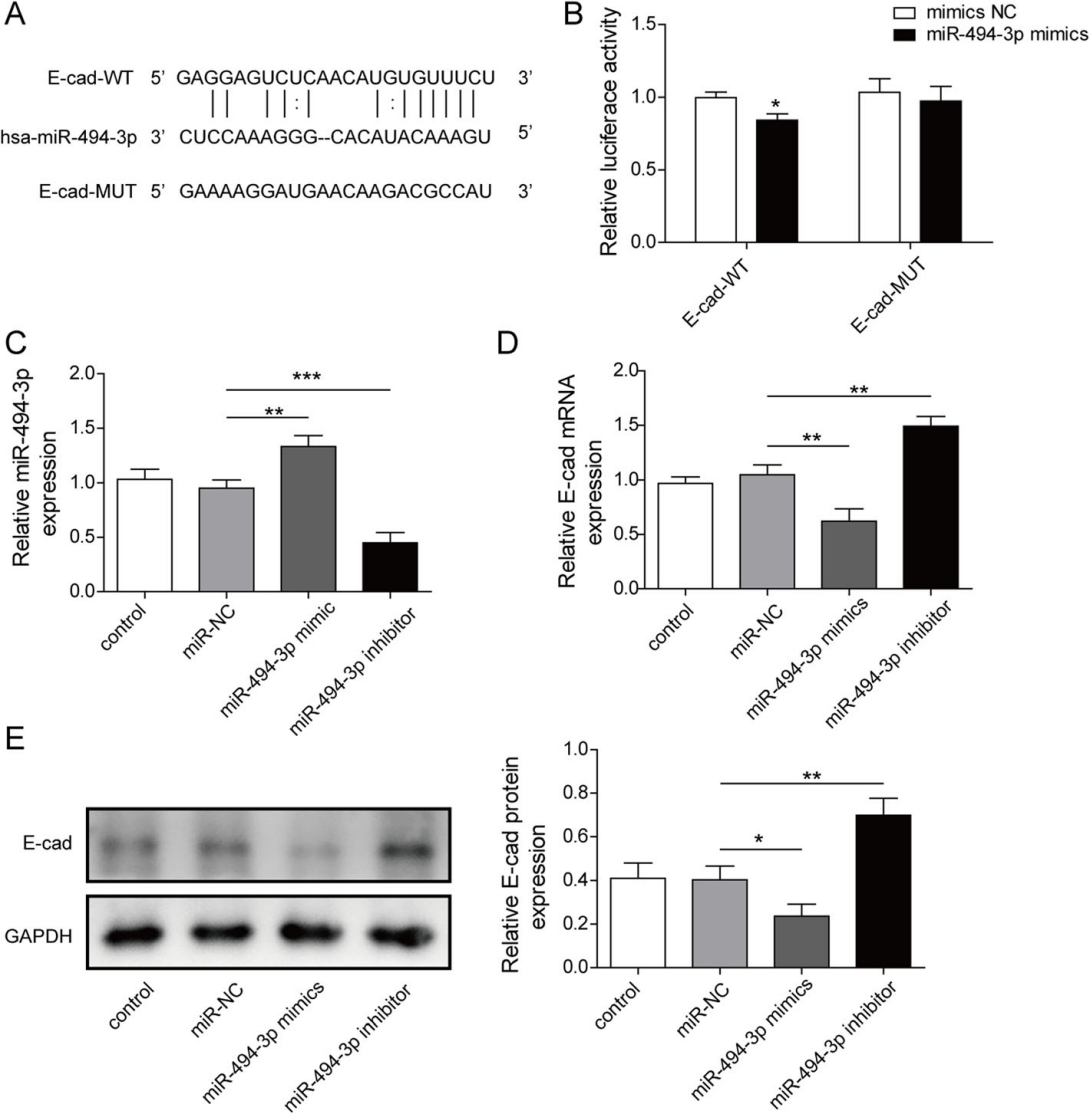
MiR-494-3p targeted to E-cadherin. **a**: The predicted binding site sequences of E-cadherin and miR-494-3p. **b**: The relative luciferase activities was assessed by luciferase reporter assay. **c**, **d**: The relative expression levels of miR-494-3p and E-cadherin mRNA were detected by qRT-PCR. **e**: The protein level of E-cadherin was measured by western blot. Results were presented as mean ± SD. *P < 0.05, **P < 0.01, ***P < 0.001

miRNAs are important in disease through targeting downstream genes. Consequently, we computed the target genes of miR-494-3p with TargetScan (http://www.targetscan.org/vert_72/). Several targets were obtained and one of them, E-cadherin, as a member of tubular adherent proteins, was eventually selected due to. its protective effect on AKI. It was reported that inhibition of the degradation of several adherent and tight junction proteins included E-cadherin could ameliorate the progression of AKI [22, 23]. Figure 5a displayed the computed binding sequences between miR-494-3p and E-cadherin. Furthermore, dual luciferase assay verified miR-494-3p mimics reduced the luciferase activity of E-cad-WT (*P* < 0.05) without changing that of E-cad-MUT (Fig. 5b), indicating that E-cadherin may be a potential target gene for miR-494-3p. Additionally, HK-2 cells were transfected with miR-494-3p mimics or miR-494-3p inhibitor to evaluated miR-494-3p. Figure 5c showed that the transfection efficiency was high. MiR-494-3p mimics down-regulated E-cadherin while miR-494-3p inhibitor showed the opposite effect in mRNA level (Fig. 5d). Also, in protein level, E-cadherin expression was decreased in miR-494-3p mimics group while was up-regulated in miR-494-3p inhibitor group (Fig. 5e). All of these data reflected that E-cadherin may be a target gene for miR-494-3p and negatively regulated by miR-494-3p.

### TUG1 regulated H/R-induced cell injury by mediating miR-494-3p/E-cadherin axis

**Fig. 6:**
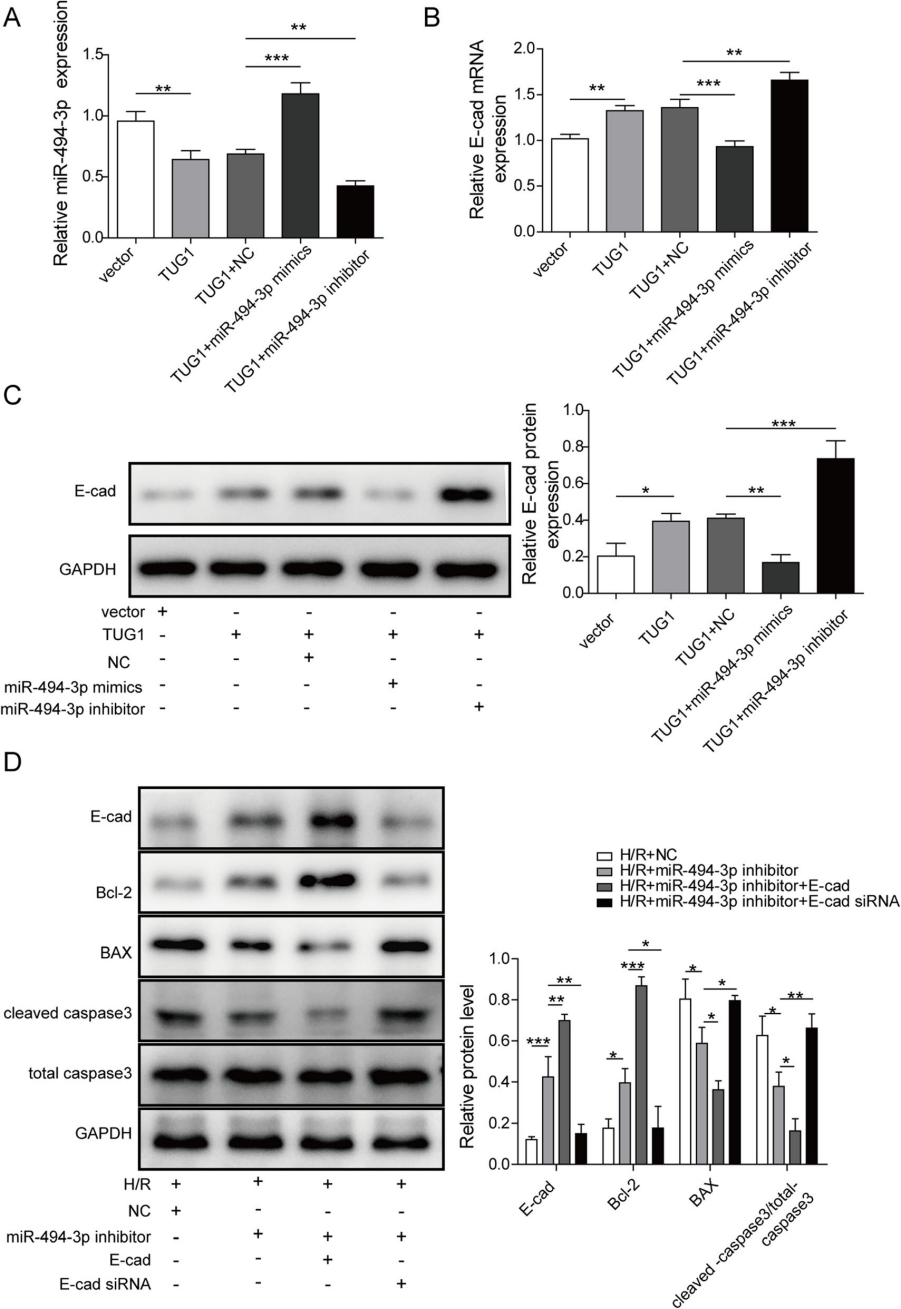
TUG1 regulated H/R-induced cell injury by mediating miR-494-3p/E-cadherin axis. **a**, **b**: The relative expression levels of miR-494-3p and E-cadherin mRNA were detected by qRT-PCR. **c, d**: The protein levels of E-cadherin and apoptosis-related proteins were measured by western blot. Results were presented as mean ± SD. *P < 0.05, **P < 0.01, ***P < 0.001

The mechanism of TUG1 in AKI was assessed in HK-2 cells. RT-PCR analysis found out in TUG1 group miR-494-3p was reduced. Concurrently, miR-494-3p mimics upregulated the expression of miR-494-3p while miR-494-3p inhibitor reduced miR-494-3p expression, indicating a high transfection efficiency (Fig. 6a). Additionally, E-cadherin mRNA was inversely associated with miR-494-3p (Fig. 6b), which is in line with the protein expression of E-cadherin (Fig. 6c). These data demonstrated that TUG1 regulated E-cadherin by mediating miR-494-3p.

Furthermore, miR-494-3p inhibitor, pcDNA E-cadherin and E-cadherin siRNA was transfected into H/R-induced HK-2 cells, respectively. MiR-494-3p inhibitor dramatically elevated the protein level of E-cadherin and Bcl-2 but reduced Bax and cleaved-caspase3. Compared to miR-494-3p inhibitor alone, E-cadherin and Bcl-2 were markedly upregulated while Bax and cleaved-caspase3 were downregulated in miR-494-3p inhibitor and pcDNA E-cadherin co-treated group. The co-transfection of miR-494-3p inhibitor and E-cadherin siRNA showed the opposite effects on all the proteins, which suggested that miR-494-3p/E-cadherin axis was associated with HK-2 cell injury (Fig. 6d). In conclude, these data speculated that TUG1 regulated H/R-induced cell injury by medicating the miR-494-3p/E-cadherin axis.

## Discussion

In our study, we found out lncRNA TUG1 was reduced both in I/R-induced mice and H/R-induced HK-2 cells. Moreover, overexpression of TUG1 effectively alleviated cell apoptosis and significantly decreased inflammatory cytokines in H/R-induced HK-2 cells. More importantly, miR-494-3p may be negatively regulated by TUG1. Further studies revealed that overexpression of TUG1 ameliorated H/R-induced cell injury by suppressing miR-494-3p. Thus E-cadherin was verified may be a target gene for miR-494-3p. Finally, our studies indicated lncRNA TUG1 might regulate H/R-induced cell injury through miR-494-3p/E-cadherin axis, which need more experiments to further attest the molecular mechanism of lncRNA TUG1 in AKI.

Extensive researches indicated that lncRNAs were increasingly important in the pathogenesis of AKI [24, 25]. For instance, lncRNA TUG1 is involved in sepsis-associated AKI by influencing cytokines production and autophagy, thus promoting cell proliferation and inhibiting cell apoptosis [13]. In line with this finding, our results showed that overexpression of TUG1 markedly relieved cell apoptosis and decreased inflammatory cytokines in H/R-induced HK-2 cells. Additionally, it has been reported that through binding to the 3′-untranslated region of miRNA, lncRNAs could influence their transcript functions, and thus impact disease development and prognosis [26, 27]. Ying Ding et al. [28] found that lncRNA MALAT1 can promote LPS-induced AKI by regulating miR-146a/NF-κB. In accordance with above studies, our results revealed miR-494-3p was negatively associated with TUG1 and overexpression of TUG1 attenuated H/R-induced HK-2 cell injury by restraining miR-494-3p.

Moreover, we identified E-cadherin may be a target gene for miR-494-3p, therefore E-cadherin was negatively adjusted by miR-494-3p in HK-2 cells. As a member of tubular adherent proteins, E-cadherin was reported to be involved in renal cell carcinoma (RCC) and renal fibrotic diseases [29, 30]. More importantly, the inhibition of Src kinase defended I/R-mediated AKI, partly due to preventing downregulation of E-cadherin [22]. Li Gao et al. also found that restoration of E-cadherin could ameliorate inflammation and cell apoptosis to attenuate the progression of cisplatin induced AKI [23]. We further found that overexpression of TUG1 can regulate E-cadherin expression by targeting miR-494-3p and overexpression or suppression of E-cadherin changed apoptosis-associated proteins in HK-2 cells transfecting with miR-494-3p inhibitor, suggesting lncRNA TUG1 might regulate H/R-induced cell injury through miR-494-3p/E-cadherin axis. In the future, we will further focus on the study of mechanism of lncRNA TUG1 in AKI and use further experiments to verify deeply the current possible conclusion.

## Conclusions

Our research indicates overexpression of the lncRNA TUG1 alleviate I/R-induced AKI by influencing miR-494-3p/E-cadherin. This may provide novel insight for the strategy of AKI treatment.

## Data Availability

All data generated or analysed during this study are included in this published article [and its supplementary information files].

## Abbreviations

AKI: Acute kidney injury
lncRNA: Long noncoding RNA
TUG1: Taurine upregulated gene 1
miR-494-3p: Microrna-494
I/R: Ischemia-Reperfusion
H/R: Hypoxia/Reoxygenation
HK-2 cell: Human kidney tubular epithelial cell
E-cad: E-cadherin
NC: Negative control
TNF-α: Tumor necrosis factor α
IL-6: Interleukin 6
IL-1β: Interleukin 1β
Bcl-2: B-cell lymphoma-2
